# Supplementary material for: Deletion of wfs1 Impairs Oligodendrocyte Precursor Cells Dorsal Distribution and Myelination Through the wfs1-hmgcs1 Axis in Zebrafish
Source: Biology (Basel). 2026 Aug 16;15(16):1402. doi: 10.3390/biology15161402 (PMC13510151; doi:10.3390/biology15161402)
Supplement: Supplementary file 1 [file biology-15-01402-s001.zip › File S1-Transcriptome sequencing data and differentially expressed genes (DEGs) in wfs1-- zebrafish at 5 dpf/2.KEGG/wfs1KOvsWT/ALL/src/dre03060.html]

dre03060


- dre:436794

- Up regulated genes

436794(1.89584033184726)

- Down regulated genes

436794\_2(-1.22283198959648)

- dre:445299

- Up regulated genes

445299\_1(2.44568101209265)

- Down regulated genes

445299(-3.89398365245409)

Close
